# Supplementary material for: Exploring Biodiversity through the Lens of Knautia arvensis Pollinators: Knautia Pollinator Walks as a Monitoring Method
Source: Insects. 2024 Jul 25;15(8):563. doi: 10.3390/insects15080563 (PMC11354427; doi:10.3390/insects15080563)
Supplement: Supplementary file 1 [file insects-15-00563-s001.zip › insects-3094684-supplementary.pdf]

Supplementary Information (SI)

Figure S1. This figure illustrates the species accumulation curve derived from the pollinator monitoring data across different sites. The x-axis represents the cumulative number of site visits, indicating the sampling effort. The y-axis shows the cumulative number of morphogroup.

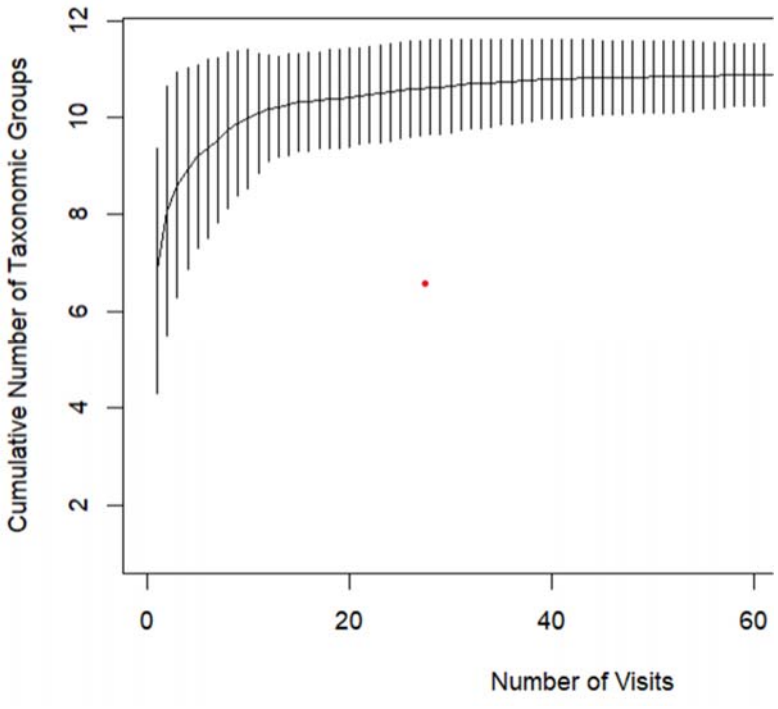

Figure S2. Boxplot illustrating the differences in species richness and density of pollinator groups between Sweden and Russia.

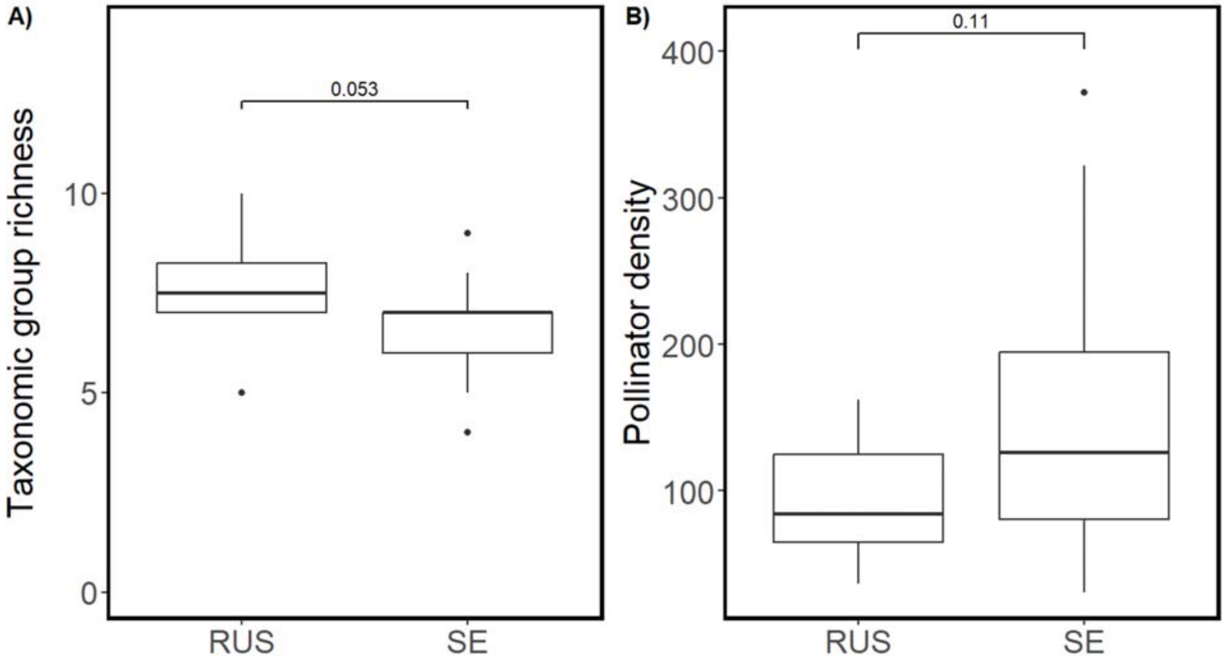
A)
Taxonomic group richness
0.053
0
5
10
RUS
SE
B)
Pollinator density
0.11
100
200
300
400
RUS
SE

Figure S3. Boxplots illustrate the differences in taxonomic group richness, density of pollinators, and species richness on sites with and without specialist bees (*A. hattorfiana, D. suripes*).

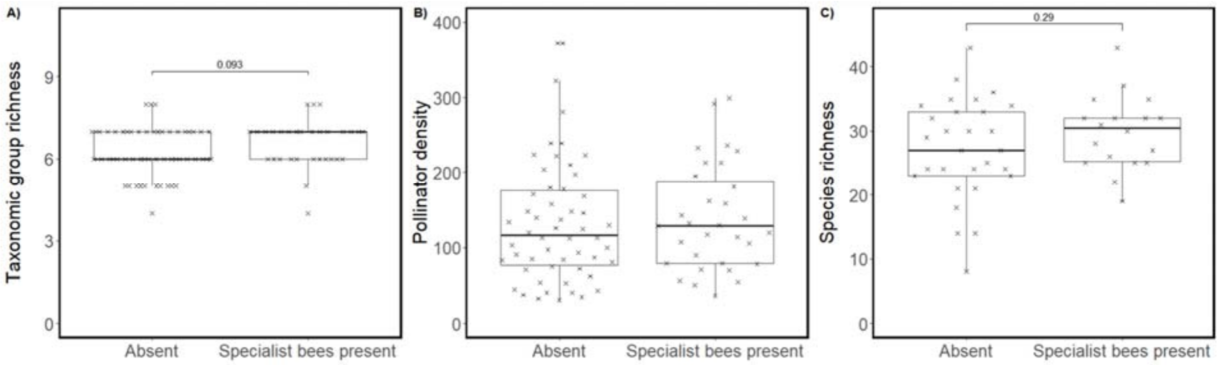

Table S1: Post Hoc comparisons of pollinator groups across two regions: Russia and Sweden

Within groups

cat1	group1	group2	n1	n2	statistic	df	p	p.adj	p.adj.signif
**Andrena hattorfiana**	**RUS**	**SE**	**8**	**77**	**2.99**	**11.9**	**0.011**	**0.011**	*
Apis mellifera	RUS	SE	8	77	1.09	7.5	0.31	0.31	ns
**Bumblebee**	**RUS**	**SE**	**8**	**77**	**-9.68**	**79.6**	**<0.001**	**<0.001**	****
**Coleoptera**	**RUS**	**SE**	**8**	**77**	**-5.41**	**66.9**	**<0.001**	**<0.001**	****
Dasypoda knautia specialist	RUS	SE	8	77	1.43	7.0	0.197	0.197	ns
**Furry Diptera**	**RUS**	**SE**	**8**	**77**	**-5.07**	**67.8**	**<0.001**	**<0.001**	****
Lepidoptera	RUS	SE	8	77	1.22	11.7	0.248	0.248	ns
**Non furry Diptera**	**RUS**	**SE**	**8**	**77**	**-6.15**	**81.4**	**<0.001**	**<0.001**	****
Other arthropod	RUS	SE	8	77	-1.23	13.5	0.238	0.238	ns
Other solitary bee	RUS	SE	8	77	2.15	7.1	0.068	0.068	ns

Table S2. Flower visitors on *Knautia arvenesis* identified to genus and/or species level found in this study. Species sorted alphabetically.

Scientific name	Auktor
Apis mellifera	[Linnaeus, 1758]
Acronicta rumicis	(Linnaeus, 1758)
Adalia bipunctata	(Linnaeus, 1758)
Adelphocoris lineolatus	(Goeze, 1778)
Adscita statices	(Linnaeus, 1758)
Aglais io	(Linnaeus, 1758)
Aglais urticae	(Linnaeus, 1758)
Alosterna tabacicolor	(De Geer, 1775)
Amara aulica	(Panzer, 1797)
Anastrangalia sanguinolenta	(Linnaeus, 1761)
Ancistrocerus trifasciatus	(Müller, 1776)
Andrena bicolor	Fabricius, 1775
Andrena coitana	(Kirby, 1802)
Andrena denticulata	(Kirby, 1802)
Andrena hattorfiana	(Fabricius, 1775)
Andrena marginata	Fabricius, 1776
Anomala dubia	(Scopoli, 1763)
Anthidium punctatum	Latreille, 1809
Anthocomus fasciatus	(Linnaeus, 1758)
Aphantopus hyperantus	(Linnaeus, 1758)
Aphis confusa	Walker, 1849
Aporia crataegi	(Linnaeus, 1758)
Argynnis paphia	(Linnaeus, 1758)
Aricia nicias	(Meigen, 1829)
Aulagromyza similis	(Brischke, 1880)
Autographa gamma	(Linnaeus, 1758)
Boloria aquilonaris	(Stichel, 1908)
Boloria selene	(Denis & Schiffermüller, 1775)
Bombus bohemicus	Seidl, 1838
Bombus campestris	(Panzer, 1801)
Bombus cingulatus	Wahlberg, 1855
Bombus hortorum	(Linnaeus, 1761)
Bombus humilis	Illiger, 1806
Bombus hypnorum	(Linnaeus, 1758)
Bombus jonellus	(Kirby, 1802)
Bombus lapidarius	(Linnaeus, 1758)
Bombus lucorum	(Linnaeus, 1761)
Bombus magnus	Vogt, 1911
Bombus muscorum	(Linnaeus, 1758)
Bombus norvegicus	(Sparre Schneider, 1918)
Bombus pascuorum	(Scopoli, 1763)

Bombus pratorum	(Linnaeus, 1761)
Bombus quadricolor	(Lepeletier, 1832)
Bombus ruderarius	(Müller, 1776)
Bombus rupestris	(Fabricius, 1793)
Bombus soroeensis	(Fabricius, 1776)
Bombus subterraneus	(Linnaeus, 1758)
Bombus sylvarum	(Linnaeus, 1761)
Bombus sylvestris	(Lepeletier, 1832)
Bombus terrestris	(Linnaeus, 1758)
Bombylius minor	Linnaeus, 1758
Brenthis ino	(Rottemburg, 1775)
Callicera aurata	(Rossi, 1790)
Carpocoris fuscispinus	(Boheman, 1850)
Carpocoris purpureipennis	(DeGeer, 1773)
Ceramica pisi	(Linnaeus, 1758)
Cerapteryx graminis	(Linnaeus, 1758)
Cetonia aurata	(Linnaeus, 1758)
Chelostoma campanularum	(Kirby, 1802)
Chelostoma rapunculi	(Lepeletier, 1841)
Chorthippus brunneus	(Thunberg, 1815)
Chromatomyia horticola	(Goureau, 1851)
Chrysanthia geniculata	Heyden, 1877
Chrysanthia viridissima	(Linnaeus, 1758)
Chrysotoxum arcuatum	(Linnaeus, 1758)
Cleopomiarus graminis	(Gyllenhal, 1813)
Coccinella septempunctata	Linnaeus, 1758
Cochylis flaviciliana	(Westwood, 1854)
Coelioxys inermis	(Kirby, 1802)
Coelioxys rufescens	Lepeletier & Serville, 1825
Colias palaeno	(Linnaeus, 1760)
Conops quadrifasciatus	De Geer, 1776
Cordylepherus viridis	(Fabricius, 1787)
Corizus hyoscyami	(Linnaeus, 1758)
Cryptocephalus sericeus	(Linnaeus, 1758)
Curculionoidea	Latreille, 1802
Dascillus cervinus	(Linnaeus, 1758)
Dasypoda hirtipes	(Fabricius, 1793)
Dasysyrphus albostriatus	(Fallén, 1817)
Dasysyrphus tricinctus	(Fallén, 1817)
Dasytes niger	(Linnaeus, 1760)
Dasytes plumbeus	(Müller, 1776)
Dolycoris baccarum	(Linnaeus, 1758)
Eilema lurideolum	(Zincken, 1817)
Eilema lutarellum	(Linnaeus, 1758)
Empis tessellata	Fabricius, 1794

Epeoloides coecutiens	(Fabricius, 1775)
Episyrphus balteatus	(De Geer, 1776)
Erebia ligea	(Linnaeus, 1758)
Eriozona syrphoides	(Fallén, 1817)
Eristalis interrupta	(Poda, 1761)
Eristalis intricaria	(Linnaeus, 1758)
Eristalis obscura	Loew, 1866
Eristalis oestracea	(Linnaeus, 1758)
Eristalis pertinax	(Scopoli, 1763)
Eristalis tenax	(Linnaeus, 1758)
Etorofus pubescens	(Fabricius, 1787)
Eupithecia centaureata	(Denis & Schiffermüller, 1775)
Fabriciana adippe	(Denis & Schiffermüller, 1775)
Gaurotes virginea	(Linnaeus, 1758)
Gonepteryx rhamni	(Linnaeus, 1758)
Halictus compressus	(Walckenaer 1802)
Halictus rubicundus	(Christ, 1791)
Harpactus tumidus	(Panzer, 1801)
Helophilus groenlandicus	(Fabricius, 1780)
Helophilus hybridus	Loew, 1846
Helophilus pendulus	(Linnaeus, 1758)
Helophilus trivittatus	(Fabricius, 1805)
Hemaris tityus	(Linnaeus, 1758)
Hesperia comma	(Linnaeus, 1758)
Hoplitis leucomelana	(Kirby, 1802)
Hoplosmia spinulosa	(Kirby, 1802)
Hylaeus brevicornis	Nylander, 1852
Hylaeus communis	Nylander, 1852
Hylaeus dilatatus	(Kirby, 1802)
Issoria lathonia	(Linnaeus, 1758)
Judolia sexmaculata	(Linnaeus, 1758)
Lasioglossum albipes	(Fabricius, 1781)
Lasioglossum calceatum	(Scopoli, 1763)
Lasioglossum fratellum	(Pérez, 1903)
Lasioglossum leucopus	(Kirby, 1802)
Lasioglossum leucozonium	(Schrank, 1781)
Lasioglossum morio	(Fabricius, 1793)
Lasioglossum zonulum	(Smith, 1848)
Lasiommata maera	(Linnaeus, 1758)
Lasiommata megera	(Linnaeus, 1767)
Leptura quadrifasciata	Linnaeus, 1758
Leucozona glaucia	(Linnaeus, 1758)
Lycaena hippothoe	(Linnaeus, 1760)
Lycaena phlaeas	(Linnaeus, 1760)
Lycaena virgaureae	(Linnaeus, 1758)

Lycophotia porphyrea	(Denis & Schiffermüller, 1775)
Macrosiphum rosae	(Linnaeus, 1758)
Maniola jurtina	(Linnaeus, 1758)
Megachile ligniseca	(Kirby, 1802)
Megachile pyrenaea	Pérez, 1890
Megachile versicolor	Smith, 1844
Megasyrphus erraticus	(Linnaeus, 1758)
Meliscaeva cinctella	(Zetterstedt, 1843)
Melitaea athalia	(Rottemburg, 1775)
Melitta haemorrhoidalis	(Fabricius, 1775)
Merodon equestris	(Fabricius, 1794)
Metrioptera brachyptera	(Linnaeus, 1761)
Miarus campanulae	(Linnaeus, 1767)
Miltochrista miniata	(Forster, 1771)
Misumena vatia	(Clerck, 1757)
Mythimna conigera	(Denis & Schiffermüller, 1775)
Mythimna ferrago	(Fabricius, 1787)
Nemophora metallica	(Poda, 1761)
Nomada armata	Herrich-Schäffer, 1839
Nomada flavopicta	(Kirby, 1802)
Nomada striata	Fabricius, 1793
Ochlodes sylvanus	(Esper, 1778)
Oedemera lurida	(Marsham, 1802)
Oedemera virescens	(Linnaeus, 1767)
Osmia leaiana	(Kirby, 1802)
Papilio machaon	Linnaeus, 1758
Pararge aegeria	(Linnaeus, 1758)
Philanthus triangulum	(Fabricius, 1775)
Phyllopertha horticola	(Linnaeus, 1758)
Pieris brassicae	(Linnaeus, 1758)
Pieris napi	(Linnaeus, 1758)
Pieris rapae	(Linnaeus, 1758)
Placochilus seladonicus	(Fallén, 1807)
Plagiognathus arbustorum	(Fabricius, 1794)
Plagiognathus chrysanthemi	(Wolff, 1804)
Polygonia c-album	(Linnaeus, 1758)
Polyommatus icarus	(Rottemburg, 1775)
Rhagium inquisitor	(Linnaeus, 1758)
Rhagonycha fulva	(Scopoli, 1763)
Rhingia borealis	Ringdahl, 1928
Scaeva selenitica	(Meigen, 1822)
Sericomyia nigra	Portschinsky, 1873
Sericomyia silentis	(Harris, 1776)
Sicus ferrugineus	(Linnaeus, 1761)
Speyeria aglaja	(Linnaeus, 1758)

Spilomyia diophthalma	(Linnaeus, 1758)
Stelis breviuscula	(Nylander, 1848)
Stelis punctulatissima	(Kirby, 1802)
Stenoptilia bipunctidactyla	(Scopoli, 1763)
Stenurella melanura	(Linnaeus, 1758)
Stenurella nigra	(Linnaeus, 1758)
Stictoleptura maculicornis	(De Geer, 1775)
Stictoleptura rubra	(Linnaeus, 1758)
Syngrapha interrogationis	(Linnaeus, 1758)
Systoechus ctenopterus	(Mikan, 1787)
Tachina grossa	(Linnaeus, 1758)
Tettigonia viridissima	(Linnaeus, 1758)
Thymelicus lineola	(Ochsenheimer, 1808)
Trachusa byssina	(Panzer, 1798)
Trichius fasciatus	(Linnaeus, 1758)
Vanessa atalanta	(Linnaeus, 1758)
Vanessa cardui	(Linnaeus, 1758)
Volucella bombylans	(Linnaeus, 1758)
Volucella inanis	(Linnaeus, 1758)
Volucella pellucens	(Linnaeus, 1758)
Xylena exsoleta	(Linnaeus, 1758)
Xysticus bifasciatus	C.L.Koch, 1837
Zygaena filipendulae	(Linnaeus, 1758)
Zygaena lonicerae	(Scheven, 1777)
Zygaena minos	(Denis & Schiffermüller, 1775)
Zygaena osterodensis	Reiss, 1921
Zygaena viciae	(Denis & Schiffermüller, 1775)
